# The Ventral Intermediate Nucleus Differently Modulates Subtype-Related Networks in Parkinson’s Disease

**DOI:** 10.3389/fnins.2019.00202

**Published:** 2019-03-11

**Authors:** Qiaoling Zeng, Xiaojun Guan, Tao Guo, Jason C. F. Law Yan Lun, Cheng Zhou, Xiao Luo, Zhujing Shen, Peiyu Huang, Minming Zhang, Guanxun Cheng

**Affiliations:** ^1^Department of Medical Imaging, Peking University Shenzhen Hospital, Shenzhen, China; ^2^Department of Radiology, The Second Affiliated Hospital, Zhejiang University School of Medicine, Hangzhou, China

**Keywords:** Parkinson’s disease, motor subtype, ventral intermediate nucleus, functional magnetic resonance imaging, granger causality analysis

## Abstract

**Background:** Posture instability gait difficulty-dominant (PIGD) and tremor-dominant (TD) are two subtypes of Parkinson’s disease (PD). The thalamus is involved in the neural circuits of both subtypes. However, which subregion of the thalamus has an influence on the PD subtypes remains unclear.

**Objective:** To explore the core subregion of the thalamus showing a significant influence on the PD subtypes and its directional interaction between the PD subtypes.

**Methods:** A total of 79 PD patients (43 TD and 36 PIGD) and 31 normal controls (NC) were enrolled, and the gray matter volume and perfusion characteristics in the thalamus were compared between the three groups. The subregion of the thalamus with significantly different perfusion and volume among three groups was used as the seed of a Granger causality analysis (GCA) to compare the causal connectivity between different subtypes.

**Results:** Perfusion with an increased gradient among the three groups (TD > PIGD > NC) in the bilateral ventral intermediate nucleus (Vim) was observed, which was positively correlated with the clinical tremor scores. The GCA revealed that TD patients had enhanced causal connectivity from the bilateral Vim to the bilateral paracentral gyrus, M1 and the cerebellum compared with the NC group, while the PIGD subtype revealed an increased causal connectivity from the bilateral Vim to the bilateral premotor cortex (preM) and putamen. Additionally, there were positive correlations between the tremor scores and a causal connectivity from the Vim to the cerebellum. The connectivity from the right Vim to the right preM and the right putamen was positively correlated with the PIGD scores.

**Conclusion:** This multilevel analysis showed that the Vim had a significant influence on the PD subtypes and that it differentially mediated the TD and PIGD-related causal connectivity pattern in PD.

## Introduction

For the past few decades, Parkinson’s disease (PD) has been categorized into posture instability gait difficulty-dominant (PIGD) and tremor-dominant (TD) subtypes, according to the predominant motor symptoms (Jankovic et al., 1990). The PIGD phenotype exhibits a later onset, more rapid deterioration of motor function, an increased risk of cognitive decline (Alves et al., 2006) and less response to levodopa compared with the TD subtype (Jankovic and Kapadia, 2001; Rajput et al., 2009). The classic mechanism of PIGD is (Kish et al., 1988) considered to be dysfunction in the striatal-thalamo-cortical (STC) circuits (Alexander et al., 1986), while that of TD is distinctively in the cerebello-thalamic-cortical (CTC) circuits (Fukuda et al., 2004; Dirkx et al., 2016). Nevertheless, the pathophysiological mechanisms underlying these disparate circuits and their clinical manifestations are not currently well understood (Helmich et al., 2012). Investigating the pathophysiological differences between the motor subtypes of PD will be a significant step toward elucidating the mechanisms underlying the distinct manifestations (Barbagallo et al., 2017) and will allow for more tailored treatment strategies.

The STC and CTC circuits share a common hub, the thalamus (Duval et al., 2016; Guan et al., 2017). The thalamus is commonly divided into seven sub-regions (Behrens et al., 2003). Only specific subregions, not the entire thalamus, are involved in the pathological mechanism of PD. Of these, the ventral intermediate nucleus (Vim) has been frequently reported (Duval et al., 2016). Deep brain stimulation (DBS) implanted in the Vim can clinically improve cerebral blood flow (CBF) and the cerebral metabolic rate (Katayama et al., 1986) with an associated improvement of tremor (Cury et al., 2017). However, this is not the case for PIGD patients (Deiber et al., 1993; Ondo et al., 1998). Additionally, CBF in the thalamus also tends to increase in PD patients with gait difficulty (Henriksen and Boas, 1985). These findings suggest that some specific thalamic nuclei play an important role in the motor phenotype of PD. However, it is not known which subregions of the thalamus play a role in the subtype of PD, and what role it plays.

Neuronal system intrinsic interaction is causal with directivity (Deshpande et al., 2011). Therefore, investigating the directional circuit about thalamus subregions would provide a new avenue for deepening our understanding of different PD subtypes. However, the traditional functional connectivity is ambiguous in terms of the underlying causal interactions. Fortunately, this can be determined by a feasible model——Granger causality analysis (GCA). GCA is a reliable method for identifying directed functional (“causal”) connectivity (Jiao et al., 2011), which has been used in some studies to explore degenerative disease pathogenesis (Florin et al., 2010, 2016; Yao et al., 2017).

Taken together, in the present study we aim to explore the core subregion of the thalamus, showing a significant influence on the PD subtypes and the directional interactions between the PD subtypes within the STC and CTC circuits. We first compare the gray matter volume and perfusion characteristics within the thalamus between the PD subtypes. Next, we compare the effective connectivity between the core subregion of the thalamic nuclei within the STC and CTC pathways in the PIGD and TD subtypes, respectively, using GCA.

## Materials and Methods

### Subjects

A total of 79 PD subjects (43 TD and 36 PIGD patients) and 31 NCs were recruited for this study from the Second Affiliated Hospital of the Zhejiang University School of Medicine. All participants were right handed. The PD patients were all diagnosed according to the United Kingdom Parkinson’s Disease Brain Bank (Hughes et al., 1992) by an experienced neurologist. Before a magnetic resonance imaging (MRI) scan and clinical assessments, the PD patients were asked to withdraw from all anti-Parkinson medications for approximately 12 h. The Unified Parkinson’s Disease Rating Scale (UPDRS), the Hoehn and Yahr disability scale (HY) and the Mini-Mental Sate Examination (MMSE) were obtained from each PD subject. Psychiatric or other neurological illnesses constituted exclusion criteria as Miroshnichenko et al. reported (Miroshnichenko et al., 2018). All subjects were free of hypothyroidism, epilepsy, drug/alcohol/nicotine abuse, and free of kidney or liver disease. The patients’ tremor scores were defined by summing items 16 and 20–21 of the UPDRS and dividing the sum by 3, and the balance and gait score were defined by adding items 13–15 and 29–30 and dividing the sum by 5. Patients were determined as TD if the ratio of the tremor score divided by the balance and gait score was ≥1.50, and as PIGD if the ratio was ≤1 (Huertas et al., 2017). The levodopa equivalent dose (LED) was calculated as formerly reported (Tomlinson et al., 2010). Every subject signed informed consent. This study was approved by the Ethics Committee of the Second Affiliated Hospital of the Zhejiang University School of Medicine.

### MRI Data Acquisition and Preprocessing

The images were acquired using a 3.0-T scanner (GE Discovery 750) with an eight-channel head coil. Foam pads were placed on both sides of the lower jaw to limit head motion. All patients were asked to keep their eyes closed and to avoid falling asleep. The anatomical data were acquired using T1-weighted sagittal images (3DMPRAGE T1, repetition time (TR) = 7.3 ms, echo time (TE) = 3.0 ms, field of view (FOV) = 260 × 260 mm^2^, matrix size = 256 × 256, slice thickness = 1.2 mm, 196 slices). Arterial spin labeling (ASL) images were acquired with a pseudocontinuous ASL sequence and background suppression (TR = 4632 ms, TE = 10.5 ms, postlabeling delay = 1.5 s, labeling duration = 1.5 s, eight interleaved spiral arms, 30 phase encoded and 512 samples at a 62.5 kHz bandwidth, slice thickness = 4 mm, NEX = 3). Axial echo-planar imaging (EPI) resting-state fMRI images were also acquired (TR = 2000 ms, TE = 30 ms, flip angle = 77°, FOV = 240 × 240 mm^2^, matrix size = 64 × 64, slice thickness = 4 mm, slices = 38, time points = 205).

The arterial spin labeling (ASL) images were preprocessed based on a voxel-wise analysis with SPM12^1^ and FMRIB Software Library (FSL) toolbox as follows: (i) every subject’s arterial spin labeling-derived perfusion map was coregistered to the CBF images; (ii) the normalization parameters produced were used to warp the perfusion images (CBF images) into the standardized space of the Montreal Neurological Institute (MNI) EPI template; (iii) normalized (unmodulated) CBF images were resliced to 2 × 2 × 2 mm^3^; (iv) the images were standardized using the whole brain mean CBF value; (v) and the images were smoothed using an 8 mm full width at half maximum (FWHM) Gaussian filter.

Voxel-based-morphometry (VBM) analyses of the structural images were performed with the VBM12 toolbox, using the default parameters and incorporating the DARTEL toolbox in the SPM 12 software. All structural images were coregistered using a linear transformation. Then, by using a unified segmentation algorithm, the resulting structural images were segmented into gray matter (GM), white matter (WM), and cerebrospinal fluid (CSF). The GM maps were affine-transformed into MNI space and further modulated to compensate for the local compression and stretching that occurs as a consequence of the warping and affine transformation. Finally, the resultant GM maps were smoothed with a Gaussian kernel with an 8 mm FWHM.

The fMRI data were preprocessed and analyzed with Dpabi^2^ and SPM12. All data were coregistered, normalized and smoothed successively (see Supplementary Material for details).

### Regions of Interest

We defined the thalamus as the region of interest (ROI) from the Automated Anatomical Labeling (AAL) template (Tzourio-Mazoyer et al., 2002). In this ROI, the perfusion parameters and gray matter volume differences were compared between the three groups. As a result, specific regions within the thalamus where there were statistical differences between the three groups were obtained. This statistically specific regions was overlaid to the Oxford thalamic atlas using the FSL toolbox (Behrens et al., 2003) to locate its specific subregion within the thalamus. This resulting subregion was also seeded for GCA analysis in the STC and CTC loops. The STC and CTC circuits were identified as the basal ganglia [globus pallidus (GP), putamen, and caudate], the motor cortex/premotor cortex, somatosensory cortex and cerebellum (Alexander et al., 1986; Zhang et al., 2015; Supplementary Figure S1).

### Causal Connectivity Between the Thalamus and the STC-CTC Circuits

Causal connectivity characterizes the direct causal effect of one brain area on another area (Deshpande and Hu, 2012). GCA is a reliable causal connectivity analytical method (Deshpande et al., 2011). It is an approach that defines causality as a tendency for the past values of a time series to improve the accuracy of predicting the future value of a time series (Park and Madsen, 2017). The basic idea is that if the previous X and Y time series can more accurately predict the current X than the previous X time series alone, then the time series Y is causally driving the time series X (Chen et al., 2009). Since there were no differences in the VBM among the groups, the seed areas were defined as the abnormal CBF areas in the group comparisons (which were located in the Vim in the Oxford thalamic atlas), with coordinates: *x* = -12, *y* = 16, *z* = 4 (left) and *x* = 12, *y* = 15, *z* = 4 (right), with a 3 mm radius. The GCA value was calculated using the REST-GCA toolkit (Zang et al., 2012) based on an ROI-wise analysis using the age and sex as covariates. A signed-path coefficient algorithm was selected to calculate the effective connectivity from the Vim to other regions in the STC and CTC circuits and from other regions in the STC and CTC circuits to the Vim.

### The Lateralization of the TD Subtype Circuits

There are hemispheric differences in the tremor-related circuit. Is the circuit in the brain of the patient with only one side of the limb tremor bilateral or unilateral? To further verify the lateralization of the TD subtype circuit, a complementary analysis was performed. Thirteen TD patients with only left limb tremor and four TD patients with only right limb tremor were collected. The brains of this four patients with only right limb tremors were then turned left and right using the FSL toolbox, and the four patients were reclassified as left limb tremors. Thus, these 17 TD patients can be considered to have tremor only on the left limb. Then explore the loop in their brains. We calculated the causal connectivity between the bilateral thalamus and STC circuit in the same manner as above. Since PIGD is dominated by axial symptoms, we did not perform an analysis of lateralization in the PIGD subtype.

### Statistics Analysis

One-way analysis of variance (ANOVA) was employed to compare the demographic, clinical information, VBM and CBF of the bilateral thalamus differences among three groups. Then, a false discovery rate (FDR) corrected *post hoc* analysis was conducted with *P* < 0.05.

Causal connectivity statistics were conducted using the random-effects model implemented in SPM12. Due to the asymmetric nature of PD, which is important in pathophysiological mechanisms, we compared the causal connectivity of the most-affected and least-affected thalamus in the STC and CTC circuits. We performed a 3 × 2 full ANOVA (full factorial design) with Group (43 TD, 36 PIGD, 31 NC) and Hemisphere (most affected, least affected) factors. Because there were no interaction effect between the Group and Hemisphere, we performed a *post hoc* analysis among the groups with the age and gender as covariates (*P* < 0.05, FDR corrected). Finally, the correlation between these variables and motor deterioration was analyzed in PD subtypes using SPSS 19 (IBM Corporation, New York, NY, United States).

## Results

### Demographic and Clinical Information

There were no statistically significant differences in gender, age, educational levels or MMSE scores among the three groups. For the PD subtypes, no statistically significant differences were found in the disease duration, total UPRDS, LED or Hoehn and Yahr scores (Table 1).

**Table 1:** Demographic characteristics.

	PIGD	NC	TD	P		
				PIGD vs. NC	TD vs. PIGD	TD vs. NC
Number of patients	36	31	43	–	–	–
Sex (male/female)	18/18	12/19	24/19	0.63	0.87	0.32
Age, years (SD)	62.11(7.03)	59.10(7.42)	62.53(8.96)	0.11	0.97	0.16
H-Ystage	2.00	–	1.82	0.00	0.11	0.00
education, years (SD)	8.42(5.72)	9.68(4.11)	7.56(4.22)	0.53	0.70	0.14
Duration, years (SD)	4.11(4.56)	–	4.60(2.76)	0.00	0.75	–
MMSE, mean (SD)	26.00(5.33)	27.35(5.45)	25.58(5.68)	0.58	0.94	0.37
Moca, mean (SD)	22.81(5.87)	23.13(6.34)	22.66(5.76)	0.97	0.89	0.79
LED, mean (SD)	249.58(262.04)	0	181.16(231.94)	–	0.33	–
Tremor scores, mean (SD)	2.36(1.71)	–	8.37(4.56)	–	0.00	–
PIGD scores, mean (SD)	4.31(2.58)	–	2.74(1.72)	–	0.00	–
UPDRS-III, mean (SD)	27.78(13.58)	0.29(0.64)	28.84(14.84)	0.00	0.92	0.00
UPDRS-total, mean (SD)	40.89(18.16)	0.29(0.64)	40.19(19.53)	0.00	0.98	0.00


*UPDRS-III, unified Parkinson’s disease rating scale part III; MMSE, mini mental state examination; oxH-Ystage, hoehn and yahr staging for Parkinson’s disease; LED, the levodopa equivalent dose. Group comparison conducted using ANOVA*.

### The Different Perfusions and Volume in the Subregion of the Thalamus

**FIGURE 1:**
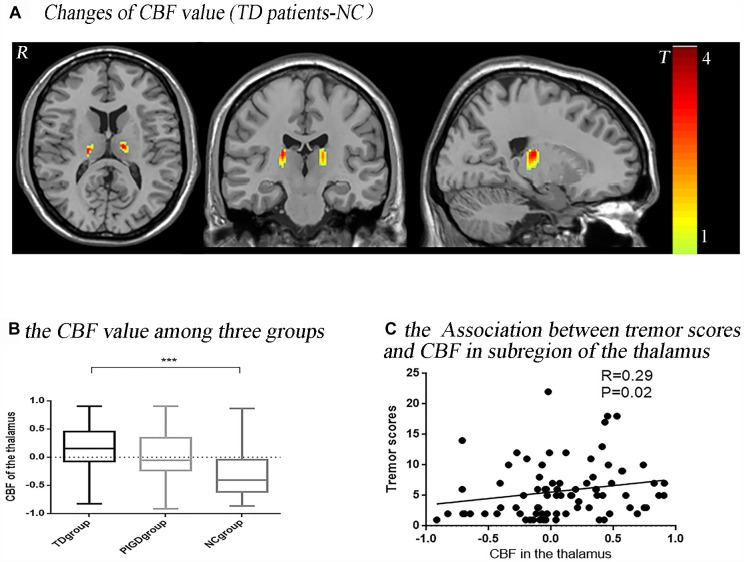
Tremor-dominant subtype showed significantly increased perfusion in the ventral intermediate nucleus (Vim) with *P* < 0.05, FDR corrected **(A)**. There was a trend of an increased CBF value in the Vim in a test threshold (*P* < 0.005, cluster size >10), especially from the NC to PIGD to TD groups **(B)**. The CBF value in this significant area (bilateral Vim) was positively correlated with clinical tremor scores **(C)**. ^∗∗∗^*P* < 0.05.

**FIGURE 2:**
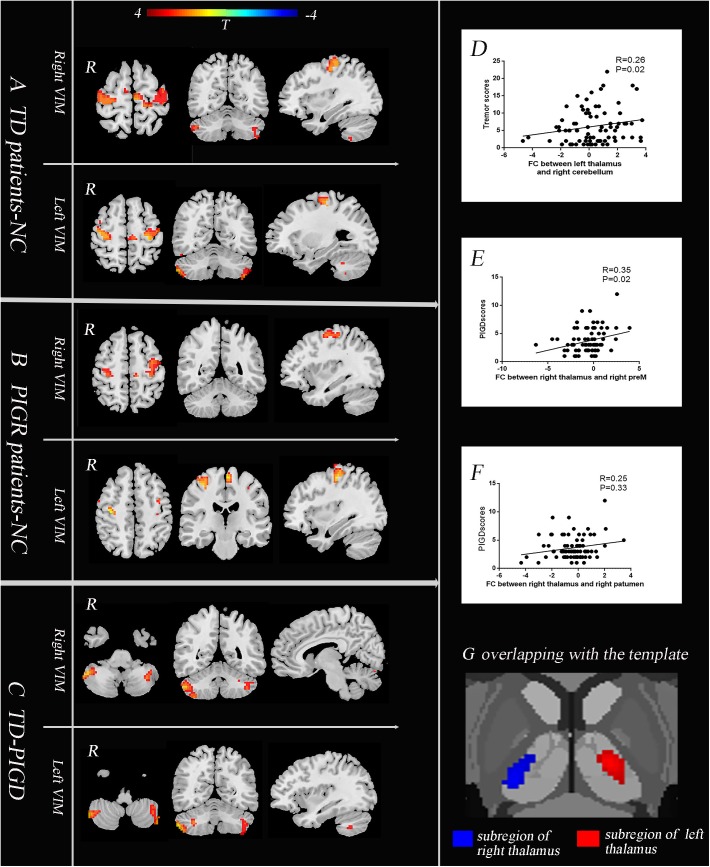
The core pathophysiology subregion of the thalamus lies in the Vim **(G)**. TD patients have enhanced causal connectivity from the bilateral Vim to the contralateral paracentral, bilateral M1 and cerebellum compared with the NC group **(A)**. The PIGD subtype revealed an increased causal connectivity from the bilateral Vim to the bilateral premotor cortex and putamen **(B)**. Directly comparing TD and PIGD, the connectivity from the bilateral Vim to the bilateral cerebellum significantly increased in TD **(C)** **(A–C)**, *P* < 0.05, FDR corrected. Correlation analysis showed that there were positive correlations between the tremor scores and the causal connectivity from the left Vim to the right cerebellum **(D)**, between the PIGD scores and causal connectivity from the right Vim to the right preM **(E)**, and between PIGD scores and the causal connectivity from the right Vim to right putamen **(F)**.

The *post hoc* analysis revealed that the TD group had significantly higher CBF values than the NC group in the bilateral thalamus (Figure 1A), with most lying in the Vim. In the left hemisphere, 92.67% (volume: 523 mm^3^) laid in the Vim, and in the right, 72.12% (520 mm^3^) laid in the Vim (Figure 2G). Even though the difference in the CBF values between the PIGD group and the NC group did not have a statistical significance after the FDR correction, there was a trend of an increased CBF value in the bilateral Vim in the PIGD group at a test threshold (*P* < 0.005, cluster size >10) (Figure 1B). Moreover, The CBF value in the Vim was positively correlated with the clinical tremor scores (*r* = 0.29, *p* = 0.02) (Figure 1C). There was no significant difference in the gray matter volume among these groups.

### The Causal Connectivity Between the Bilateral Vim and STC-CTC Circuits

We then explored the causal connectivity patterns of the Vim to the STC and CTC circuits in the PD subtypes. The group effects are shown in Figure 2. First, TD patients showed enhanced causal information from the bilateral Vim to the bilateral paracentral gyrus, M1 and cerebellum compared to the NC group (Figure 2A). There were no significant group differences between the Vim and basal ganglia in the TD group. Second, in PIGD patients, the bilateral Vim input a significantly increased flow to the bilateral premotor cortex (preM) and putamen (Figure 2B) compared to the NC group. Crucially, these effects were not observed in the caudate or GP. Third, when directly comparing the TD and PIGD groups, the information flow from the bilateral Vim to the bilateral cerebellum significantly increased in the TD patients (Figure 2C). There were no significant differences in the causal connectivity from the STC and CTC circuits to the Vim.

Furthermore, the tremor-related clinical scores were positively correlated with a causal connectivity from the left Vim to the right cerebellum (Figure 2D). The causal connectivity from the right Vim to the right putamen and right preM was positively correlated with the clinical PIGD scores (Figure 2E,F).

### Laterality of the Tremor-Related Circuit

**FIGURE 3:**
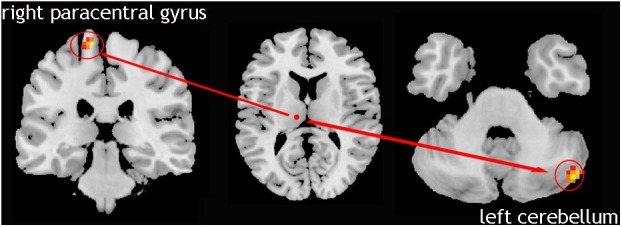
Left lateral limb-affected TD patients; showed increased causal connectivity from right Vim to left cerebellum and the right paracentral gyrus was increased.

We compared 17 TD patients who can be seen as only left lateral limb-affected patients to the NC group. The result showed that in these patients, the causal connectivity from the right Vim to the left cerebellum and right paracentral gyrus was increased (Figure 3). This analysis confirmed the laterality of the tremor-related circuit. That is, one side of the limb tremor was due to contralateral Vim disorder, and the disordered Vim enhanced the flow of information to the cerebellum on the same side of the affected limb and contralateral motor cortex.

## Discussion

**FIGURE 4:**
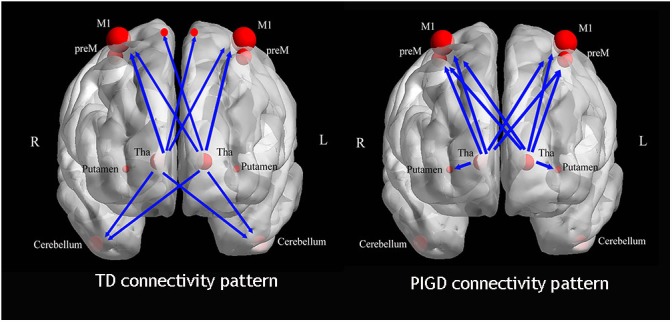
The different causal connectivity patterns in the PD subtypes.

There were three main findings. First, as a subregion of the thalamus, the Vim exhibited significantly increased perfusion in the TD subtype. This change was positively correlated with the clinical tremor scores. There was an increased gradient of the CBF value among the three groups (TD > PIGD > NC). Second, the TD and PIGD patients displayed different causal connectivity patterns (Figure 4). In TD patients, the Vim had enhanced causal connectivity with the bilateral paracentral gyrus, M1 and the cerebellum, while the causal connection from the Vim to the cerebellum was positively correlated with the tremor score. The PIGD patients showed an increased causal connectivity from the bilateral Vim to the bilateral preM and putamen. These changes were positively correlated with the PIGD scores. Third, we confirmed the TD-related circuits are from the Vim to the contralateral cerebellum and paracentral gyrus. Together, our outcomes support the presence of an underlying pathophysiological discrepancy between the PD motor subtypes.

### Perfusion in the Vim

Past studies found that PD patients exhibited significantly higher CBF in the thalamus (Hsu et al., 2007), especially in TD patients. Furthermore, the CBF alteration could be a biomarker distinguishing TD patients from essential tremor (Song et al., 2014). On that basis, we further found that almost all significant areas of the thalamus lie in the Vim. Its perfusion was significantly increased in the PD subtypes without a significant structural alteration, and these perfusion values showed a close relationship with the tremor scores. In 1-methyl-4-phenyl-1.2.3.6-tetrahydropyridine (MPTP) primate model of parkinsonism, the GP is an important cause of tremor triggering (Rivlin-Etzion et al., 2008; Kammermeier et al., 2016) due to increased synchrony among neurons (Bergman et al., 1998). Anatomically, the Vim received an afferent projection from GP, and then explosively emphasized signals (Helmich et al., 2012). Hence, the Vim was implicated in generating the supraspinal components of tremor (Duval et al., 2016). From our results, the Vim may be a “key nodal point” in both the STC and CTC circuits, affecting both PD subtypes.

### The Vim Nucleus in the Tremor-Related Causal Connectivity Pattern

Our findings indicate that the enhanced causal connectivity from the Vim to M1 and cerebellum is associated with parkinsonian tremor. In the validation, we identified that these changes coincided with hemisphere-affected TD patients.

The cerebellum is a vital hub in this circuit. It plays a critical role in parkinsonian tremor amplitude modulation (Helmich et al., 2011). In the MPTP monkey mode, improved motor symptoms are associated with the mean discharge rates of neuron activity in the cerebellar receiving areas of the motor thalamus (Vitek et al., 2012). In human, increased metabolism in the cerebellum is associated with parkinsonian tremor and its metabolism is reduced by the DBS of Vim (Mure et al., 2011). The cerebellum receives input from the posterior thalamus and then relays it back to the thalamus (Iwata and Ugawa, 2005). In the present study, the causal influence from the Vim to the cerebellum was increased in TD patients. This causal connectivity becomes more strengthened as the tremor becomes more severe. This result could imply that the Vim exerts its influence on cerebellum-stimulating activity. Notably, an enhanced influence from the Vim to the cerebellum was not found in PIGD patients. This elucidates that the Vim-cerebellum topology may be a characteristic pattern in the TD population.

Additionally, M1 is classically viewed as direct cortical selecting the muscles and force for executing an intended movement (Yao et al., 2017). The Vim nucleus has tight fiber connections with M1. The DBS of the Vim could diminish the metabolism in M1, which further advocates the pathology of the Vim to M1 in TD patients (Fukuda et al., 2004). In this study, the information flow from the Vim to M1 was enhanced in TD patients. This implies that the motor impairments in TD subtypes could be due to an abnormal strengthening output from the Vim to the motor cortex in addition to striatal pathology. Taken together, our results indicate that parkinsonian tremor is peculiarly mediated by a flow from the Vim to the cerebellum, where the dysfunction of the Vim may lead to disruption in M1 via the Vim-M1 circuit.

### The Vim Nucleus in the PIGD-Related Causal Connectivity Pattern

The pathological hallmark in PD is the progressive deficiency of dopamine within the substantia nigra and striatum (Jellinger, 1999). As the main component of striatum, the putamen has been regarded in the pathophysiology of motor impairment (Jellinger, 2012). In the non-human primate model of Parkinson’s disease, increased metabolism in the putamen is a characteristic topography (Ma et al., 2012). The putamen also play an important role in defining the PD motor subtypes (Vervoort et al., 2016). Single photon emission computed tomography studies found that patients with worse rigidity had more pronounced dopaminergic loss in the posterior putamen (Eggers et al., 2011). A lower putamen volume was linked with a higher (worse) instability gait score (Rosenberg-Katz et al., 2016). In the present study, we further found that the Vim input a significantly increased flow to the putamen in PIGD patients. Since the putamen is the major input structure of the basal ganglia and receives afferents from the thalamus (Braak et al., 2006), we suspect that the enhanced connectivity from the Vim to the putamen may be a feedback mechanism of dopaminergic loss. To balance the dynamic equilibrium between the striatum and thalamus, the Vim enhanced the feedback flow to the putamen after the decreased input from the striatum due to dopaminergic loss. Additionally, this causal connectivity was correlated with the severity of the PIGD scores, which emphasized the relation between the PIGD motor impairments and the feedback flow from the Vim to the putamen. These findings provide further evidence of the role of the putamen in PD subtypes.

Recently, some researchers have held the notion that the PIGD and TD could simply be different stages of PD (Nutt, 2016), since some TD patients evaluated at onset were predominantly PIGD in more advanced stages (Hershey et al., 1991). However, our results support the notion that the PIGD and TD motor phenotypes could not be accounted for by differences in disease stage or duration. In the present study, there were no significant differences in the duration, LED, HY stage, or UPDRS total scores for the different phenotypes. However, they expressed different causal connectivity patterns. Therefore, our findings support the existence of TD and PIGD subtypes.

There are some limitations in the present study. Though our samples are larger than many other prior reports, larger samples are required to enhance the power of future studies. Another limitation is that all patients were scanned in a practical off-state at 12 h of anti-parkinsonism medication withdrawal. While the dopamine agonists could have lasting effects, we did not find a significant difference in the LED between the different subtypes, which means the lasting effects may make no difference to the PD subtypes in our population.

In conclusion, the present study showed that the Vim may be a “key nodal point,” affecting both PD subtypes. The Vim is a causal flow hub of the STC and CTC circuits. A differential causal connectivity pattern exists in TD and PIGD-related networks, which is related to behavioral heterogeneity in PD. Our findings are helpful for explaining the existence of PD subtypes that are interrelated to TD and PIGD manifestation in PD.

## Data Availability

All datasets generated for this study are included in the manuscript and/or the Supplementary Files.

## Author Contributions

QZ, XG, and GC conceived the study and designed the protocol. QZ, TG, JLYL, and CZ performed the experiments. QZ, XL, ZS, and PH conducted the statistical analyses. QZ wrote the first draft of the manuscript. MZ and GC interpreted the study findings and reviewed the manuscript. All authors read and approved the manuscript.

## Conflict of Interest Statement

The authors declare that the research was conducted in the absence of any commercial or financial relationships that could be construed as a potential conflict of interest.

## Footnotes

1 http://www.fil.ion.ucl.ac.uk/spm/software/spm12

2 http://www.restfmri.net
