# Supplementary material for: The Ventral Intermediate Nucleus Differently Modulates Subtype-Related Networks in Parkinson’s Disease
Source: Front Neurosci. 2019 Mar 11;13:202. doi: 10.3389/fnins.2019.00202 (PMC6421280; doi:10.3389/fnins.2019.00202)
Supplement: Supplementary file 1 [file Data_Sheet_1.docx]

Supplementary Material

Ventral intermediate nucleus differently modulates subtypes -related networks in Parkinson's disease

**Qiaoling Zeng^1^, Xiaojun Guan^2^, Tao Guo ^2^, Jason C. F. Law Yan Lun^2^, Cheng Zhou ^2^, Xiao Luo^2^, ZhujingShen^2^, Peiyu Huang^2^ , Minming Zhang^2^*，Guanxun Cheng^1^***

^1^ Department of Medical Imaging, the Peking University Shenzhen Hospital, Shenzhen, China.

^2^ Department of Radiology, the Second Affiliated Hospital, Zhejiang University School of Medicine, Hangzhou, China.

*** Correspondence:**

Prof. Minming Zhang, MD, PhD E-mail: [zhangminming@zju.edu.cn](mailto:zhangminming@zju.edu.cn)

Prof. GuanXun Cheng, MD, PhD E-mail: chengguanxun@outlook.com

# Pre-processing and analysis of images

## The arterial spin labelling (ASL) image preprocessing

The arterial spin labeling (ASL) images were preprocessed based on a voxel-wise analysis with SPM12 (<http://www.fil.ion.ucl.ac.uk/spm/software/spm12>) and FMRIB Software Library (FSL) toolbox as follows: (i) every subject’s arterial spin labeling-derived perfusion map was coregistered to the cerebral blood flow images (CBF); (ii) the normalization parameters produced were used to warp the perfusion images into the standardized space of the Montreal Neurological Institute (MNI) EPI template; (iii) normalized (unmodulated) CBF images were resliced to 2 x 2 x2 mm^3^; (iv) the images were standardized using the whole brain mean CBF value; (v) and the images were smoothed using an 8 mm (FWHM) Gaussian filter. To minimize motion-related artifact we removed images if framewise displacement exceeded 0.9 mm^(^[^Stewart et al., 2015^](#_ENREF_5)^)^ and two subject’s data was excluded from further analysis.

## Structural image analysis

Voxel-based-morphometry (VBM) analyses of the structural images were performed with the VBM12 toolbox, using the default parameters and incorporating the DARTEL toolbox in the SPM 12 software. All structural images were coregistered using a linear transformation. Then, by using a unified segmentation algorithm, the resulting structural images were segmented into gray matter (GM), white matter (WM) and cerebrospinal fluid (CSF). The GM maps were affine-transformed into Montreal Neurological Institute (MNI) space and further modulated to compensate for the local compression and stretching that occurs as a consequence of the warping and affine transformation. Finally, the resultant GM maps were smoothed with a Gaussian kernel with an 8 mm full width at half maximum (FWHM).

## FMRI data preprocessing

Dpabi ([http://www.restfmri.net](http://www.restfmri.net/forum/)) and SPM12 software packages (<http://www.fil.ion.ucl.ac.uk/spm/software/spm12>) were used for fMRI data preprocessing. The first 10 volumes of the functional time series were discarded for the signal to reach equilibrium. The remaining195 fMRI images were corrected for within-volume slice-acquisition time differences. They were then realigned to correct for interscan head motion. The images from three patients were excluded because their head motion exceeded either 2 mm in displacement or 2° in rotation. Head movements during scanning did not differ among the TD, PIGD and NC groups. Then, the functional images were normalized to the Montreal Neurological Institute (MNI) EPI template and were resampled to a 3x3x3 mm isotropic resolution. This was followed by temporal filtering (0.01 Hz < f < 0.08 Hz) to remove the effects of high-frequency physiological and low-frequency drift noise. Finally, the linear trend was removed.

# Regions of Interest

We defined the thalamus as a region of interest (ROI) from an Automated Anatomical Labeling (AAL) template ([Tzourio-Mazoyer et al., 2002](#_ENREF_6)) for the perfusion and gray matter volume assessment. After comparing the perfusion parameter (cerebral blood flow, CBF) and the gray matter volume of the thalamus among different groups, we overlapped the significantly altered brain areas to the Oxford thalamic atlas using the FSL toolbox ([Behrens et al., 2003](#_ENREF_2)), which divided the thalamus into seven subregions.

The STC and CTC circuits were identified on the basal ganglia (globus pallidus, putamen, and caudate), the motor cortex/premotor cortex, somatosensory cortex and the cerebellum (MC–CBLM) according to prior studies ([Alexander et al., 1986](#_ENREF_1); [Fukuda et al., 2004](#_ENREF_4); [Dirkx et al., 2016](#_ENREF_3)) from AAL template (Suppl. Figure 1).

*
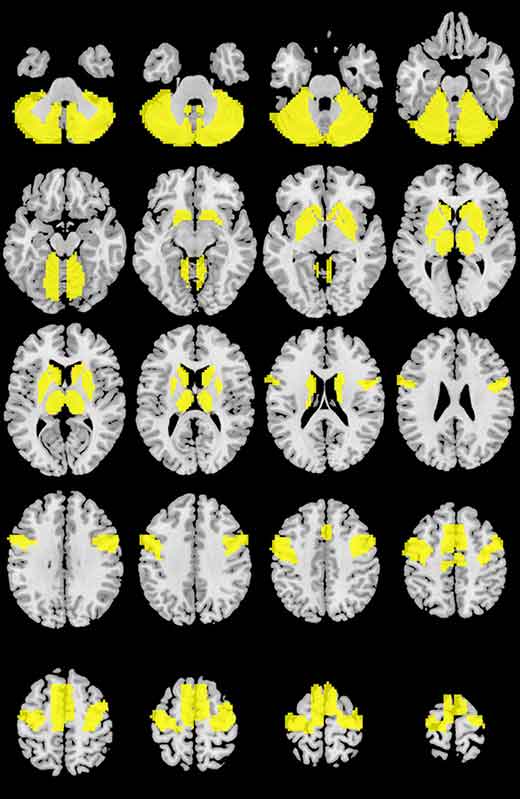
*

**Supplementary Figure 1: the STC and CTC circuit**

Reference

Alexander, G.E., DeLong, M.R., and Strick, P.L. (1986). Parallel organization of functionally segregated circuits linking basal ganglia and cortex. *Annu Rev Neurosci* 9**,** 357-381. doi: 10.1146/annurev.ne.09.030186.002041.

Behrens, T.E., Johansen-Berg, H., Woolrich, M.W., Smith, S.M., Wheeler-Kingshott, C.A., Boulby, P.A., et al. (2003). Non-invasive mapping of connections between human thalamus and cortex using diffusion imaging. *Nat Neurosci* 6(7)**,** 750-757. doi: 10.1038/nn1075.

Dirkx, M.F., den Ouden, H., Aarts, E., Timmer, M., Bloem, B.R., Toni, I., et al. (2016). The Cerebral Network of Parkinson's Tremor: An Effective Connectivity fMRI Study. *J Neurosci* 36(19)**,** 5362-5372. doi: 10.1523/JNEUROSCI.3634-15.2016.

Fukuda, M., Barnes, A., Simon, E.S., Holmes, A., Dhawan, V., Giladi, N., et al. (2004). Thalamic stimulation for parkinsonian tremor: correlation between regional cerebral blood flow and physiological tremor characteristics. *Neuroimage* 21(2)**,** 608-615. doi: 10.1016/j.neuroimage.2003.09.068.

Stewart, S.B., Koller, J.M., Campbell, M.C., Perlmutter, J.S., and Black, K.J. (2015). Additive global cerebral blood flow normalization in arterial spin labeling perfusion imaging. *PeerJ* 3**,** e834. doi: 10.7717/peerj.834.

Tzourio-Mazoyer, N., Landeau, B., Papathanassiou, D., Crivello, F., Etard, O., Delcroix, N., et al. (2002). Automated anatomical labeling of activations in SPM using a macroscopic anatomical parcellation of the MNI MRI single-subject brain. *Neuroimage* 15(1)**,** 273-289. doi: 10.1006/nimg.2001.0978.
